# Enabling Communication in Emergency Response Environments

**DOI:** 10.3390/s120506380

**Published:** 2012-05-14

**Authors:** Roberto G. Aldunate, Klaus Nicholas Schmidt, Oriel Herrera

**Affiliations:** 1 Department of Computer Science, Catholic University of Temuco, Temuco, Chile; E-Mail: oherrera@uct.cl; 2 Applied Research Associates, Inc., Champaign, IL 61820, USA; 3 Department of Technology, Illinois State University, Normal, IL 61761, USA; E-Mail: klaus.schmidt@ilstu.edu

**Keywords:** emergency response, augmented reality, natural interface, intuitive interface

## Abstract

Effective communication among first responders during response to natural and human-made large-scale catastrophes has increased tremendously during the last decade. However, most efforts to achieve a higher degree of effectiveness in communication lack synergy between the environment and the technology involved to support first responders operations. This article presents a natural and intuitive interface to support Stigmergy; or communication through the environment, based on intuitively marking and retrieving information from the environment with a pointer. A prototype of the system was built and tested in the field, however the pointing activity revealed challenges regarding accuracy due to limitations of the sensors used. The results obtained from these field tests were the basis for this research effort and will have the potential to enable communication through the environment for first responders operating in highly dynamical and inhospitable disaster relief environments.

## 1. Introduction

In the aftermath of a large-scale natural or manmade disaster, the effectiveness of first responders (FRs) continues to play a key role in determining the time required to put the urban infrastructure back to normal operations. A variety of approaches for disaster relieve have been researched in the past, generating and determining coordination and communications problems at both machine-to-machine and human-to-human layers [1]. In a permanent effort to mitigate the impact of disasters by improving the performance of field FRs, new technology, or novel uses of existing technologies, are constantly explored by engineers and scientists [2,3]. Traditional communication and coordination platforms and tools seem to have limited capability to enable effective collaboration among field FRs, given the non-natural and intuitive human-to-machine and human-to-human interfaces. For example, Geographic Information Systems (GIS) and location based applications seem suitable for desktop computers or some mobile communication devices utilized on command posts, but are of limited applicability for firefighters or rescue crews trying to locate people from a collapsed structure, where their hands and their cognitive resources are focused on tasks strongly tied to ongoing activities in a chaotic building infrastructure. This article presents a novel approach for the development of technological support for FRs conducting field operations. This approach is based on enabling basic language capabilities [4] and communication via the environment (*i.e.*, Stigmergy) [5] by means of an intuitive and natural human-to-human interaction using a robust and distributed machine-to-machine interface. Stressing this problem with traditional augmented reality approaches would result in flooding the FR's vision with digital data (*i.e.*, text and icons), which can be distractive to the rescue team. Hence, a novel interface concept is required. In particular an interface that is equally fast and reliable in natural and in manmade hazards that allows FRs to act quickly, precisely and without harming themselves or putting their colleagues at risk. Coordinating rescue missions during disasters are becoming more challenging as more people get involved and require coordination. Emergent response groups that are characterized by a sense of great urgency and high levels of interdependence need to operate in environments that are constantly changing as new information arrives about rescuing victims. This volatility of information combined with the adapting of a fast changing environment, or at least the fast changing perception of an environment, need to adapt to unstable task definitions, allow for flexible task assignments, and coordinate possibly multiple, simultaneous and conflicting purposes [6].

The rest of this article is structured as follows. The system design section introduces the system components and their relationships, both geared towards enabling intuitive and natural communication through the environment. The section on prototype development and testing shows the prototyping effort conducted to proof the concept stated in this research effort. The section on related work presents related relevant work of this initiative, with emphasis on augmented reality. A discussion of some of the main issues regarding performance of the system presented in this article will follow and conclusions by the researchers are presented followed by the references.

## 2. System Design

**Figure 1.**
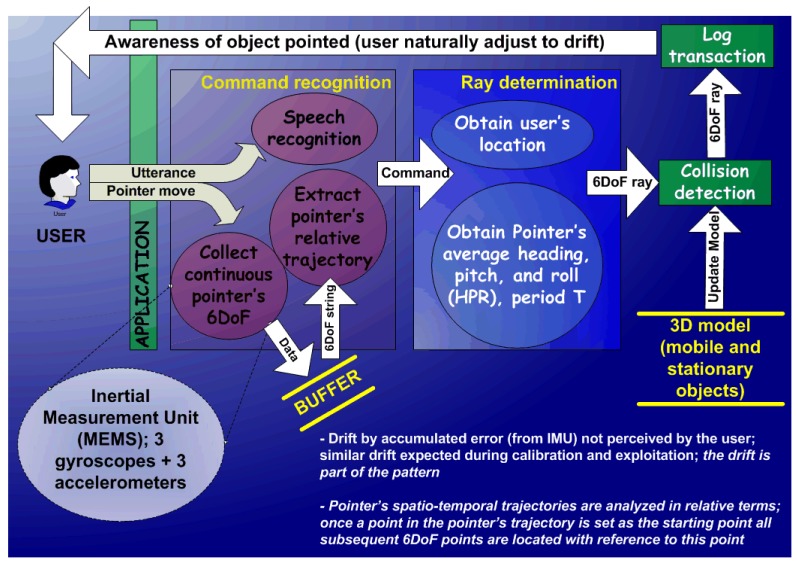
Functional flow diagram for natural and intuitive interface for emergency response environments: (**a**) the FR (user) activates the pointer (pointer's movement pattern recognition) in selection mode; (**b**) location of FR is determined, if s/he is not moving, set stationary location on 3D model (eliminates its impact on further pointing drift); (**c**) orientation of pointer (electronic compass) is obtained (6DoF is complete); (**d**) 3D model is aligned with the observed real world object; collision detection determines object pointed to; (**e**) render on display what is being pointed to (feedback); (**f**) the FR observes real world overlaid with the object pointed to in a 3D model (e.g., contour of facility); (**g**) and the FR naturally adjusts the pointing drift. The compass's absolute precision becomes irrelevant while it is oriented towards the aimed target (analogous to a person pointing an object with her finger).

The concept behind this research effort focuses on finding ways to *naturally* pointing to objects and annotating or retrieving information necessary to provide solutions in a chaotic environment (see Figure 1). The central piece of the pointing component is a pointer, attachable to any part of the FR's body, which operates on a *naturally* augmented space. This space is defined by the physical world observed by the FR, which can be implemented with a wearable device like a mobile phone or a head-up display. The *natural* attribute is a key concept that scales analog data to a virtual reality environment, and does not require icons or textual information (e.g., the contour of the ‘virtual’ facility overlays with the observed facility). As teams of FRs operate in a common geographic area, they can then interact in both a synchronous or asynchronous manner through this overlaid physical landscape, rather than working in a digital representation of it.

This paper presents an effort to integrate several available technologies to facilitate the communication and coordination a cohesive emergency response system for chaotic environments. These technologies include a wireless communication platform such as 3G/4G cellular networks or WiFi/WiMax short range data packet networks, a 3D terrain models of the surroundings (optional), a location service such as the Global Positioning System (GPS), and camera-enabled head-up display (HUD) as used for both commercial and military field operations.

Our preliminary observations on how a person points to an object in her environment suggest studying several additional aspects: (a) individuals tend to remain in a stationary position while pointing to some objects, allowing discarding the component of drift. What remains is only the angular component, hence pointing accuracy will rely almost entirely on the compass's precision (*i.e.*, how stable are the compass's readings?); (b) when a person points to something with her finger she will orient her finger towards the target in relative terms only because a human's vision and arms cannot be aligned in a straight line (in opposition to the alignment between vision and the gun's aim when firing a gun). *The finger pointing activity incorporates a natural drift*. Finally, (c) the *natural drift* grows with the distance between the person and the targeted object. This concept is easy to easy to demonstrate. If a person closes one eye and adjusts the tip of the finger with an imaginary ray between one's eye and the target and then closes the eye viewing the same object through the other eye, the finger will not be pointed directly to this very object. Therefore what emerges as important is not what the finger is pointing at, but what the person *sees* the finger is pointing at. Put in other words, once analogous feedback of the orientation of the pointer (e.g., a ray or a virtual finger) is provided to a person, she seems to naturally adjust to small natural drift as long as this drift is constant.

**Figure 2.**
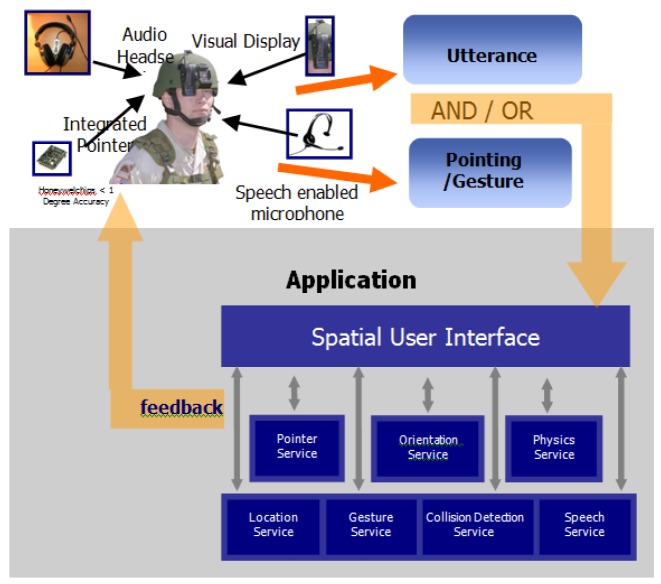
Interaction diagram describing how the interface works and the underlying agent-based architecture.

**Figure 3.**
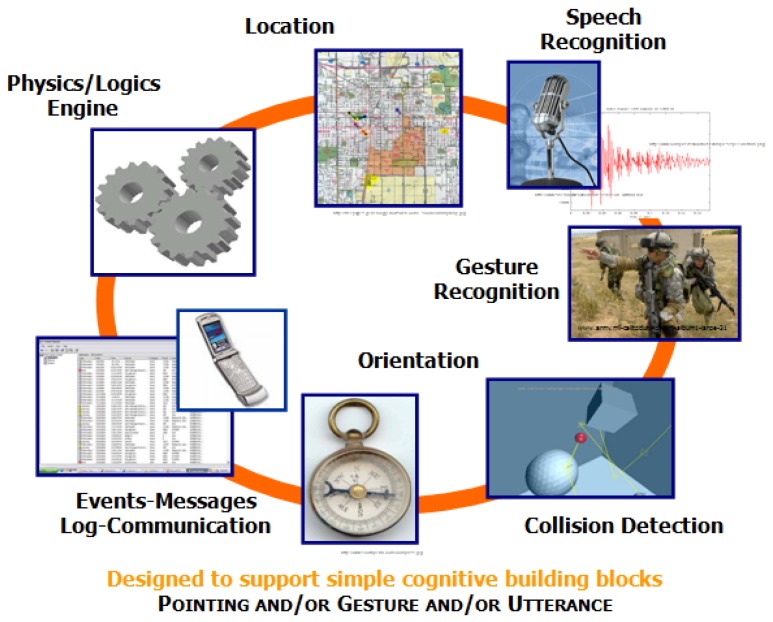
Schematic description of components for the natural an intuitive interface-technological components of the platform.

In addition to pointing drift, there are two key components for the design of the natural and intuitive system: the gesture recognition capability and the integration of the technologies comprising the system such as location, orientation, and pointer-object collision. While Figure 2 shows how this integration was achieved through the development of an agent-based architecture, Figure 3 shows a schematic description of components for the natural an intuitive interface displaying technological components of the platform. These two components are described in the following paragraphs.

### Customizable Gesture Recognition

The principle for this service is simple; the FR defines his/her own patterns for the pointer's operation; the “click” gesture. These gesture recognition building blocks (e.g., pointing, selecting what is pointed to, directing the attention of peers to what is pointed to) require input by subject matter experts in emergency management operations.

The core of gesture recognition is finding matches between the pointer's trajectories and pre-defined trajectories, where a trajectory is a set of six degree of freedom (6DoF; XYZ location + heading, pitch, and roll) points in space and time. This recognition can be as simple as dot-products between the current and pre-defined trajectory vectors to more complexes pattern recognition mechanisms such as hidden Markov models, particle filtering, and neural networks. The neural networks solutions have the potential to better interact with incomplete and uncertain data (*i.e.*, error in data gathering) [7]. We evaluated various recognition algorithms [8,9], their required training, accuracy, reliability and dependability under various conditions. Then, we implemented several of those algorithms exploring changes such as the number of layers and firing thresholds, and created variations and combinations of them in the prototype. An additional neural networks training phase supports the customizable goal for gesture recognition; every user tries and defines her or his hand (as a point) gestures for a given command. For example, we defined simple horizontal and vertical oscillatory movements to represent a PC Mouse simple and double “clicks”. Given current Commercial Off The Shelf (COTS) electronic compass and accelerometer technology, with output at the range of a couple of Hertz, we were able to gather the data necessary to obtain hand trajectories and gestures matching in space and time with 90% reliability. We considered this reliability value high enough for the purpose of this research and development effort, even though better results have been reported in literature [10].

### Agent-based Architecture

An agent-based architecture enables the system to upgrade and improve COTS equipment - like see-through visors-as it becomes available while allowing the system to be scalable, more fault tolerant, and achieve higher precision performance. Likewise, customized components and equipment can be developed or implemented to meet additional emergency response environment requirements. For the system implementation at hand, the following *ad hoc* components were built:

- A collision detection engine specifically tuned to satisfy near-real-time processing
- A gesture recognition service
- A log service to record events supported by the system (and to support memory-based activities like operations analysis for training purposes)
- A physics/logics engine to model the effects of events in simulated environments (oriented for training purposes)
- This agent-based architecture, which combine all the different components in a scalable and fault tolerant manner

The agent-based architecture allows the application to perfectly fit into centralized or distributed (or any combination of them) computer networks. In a distributed peer-to-peer setting, the peer originating a pointing event will broadcast a message containing the origin and direction of the pointing ray. Each peer (processing device) receiving such a message will determine its location to ascertain whether or not it is on the ray's trajectory. If so, it will send a message back to the ray's generator. Then, the generator processes the acknowledged collision to determine and update the state of the distributed system, e.g., target device identified for marking/messaging purposes. On the other hand, in a centralized setting, the services reside on servers, *i.e.*, collision detection is performed by a server aware of all movements and characteristics of the objects in the 3D model.

## 3. Prototype Development and Testing

**Figure 4.**
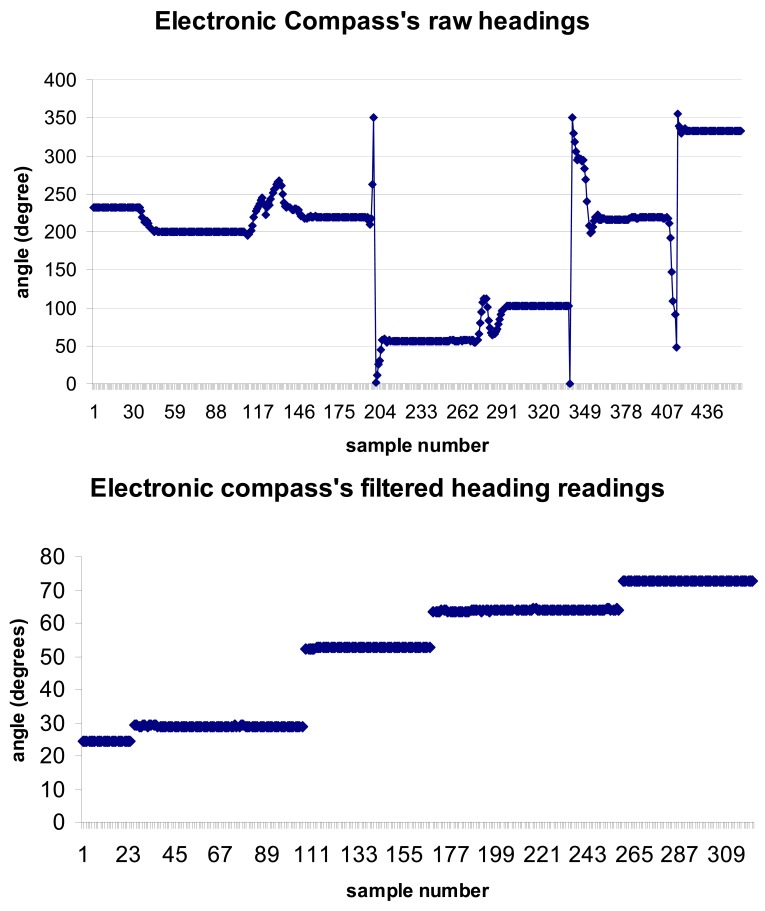
Heading readings obtained with a Honeywell HMR3300 electronic compass: raw readings (perturbations correspond to quick changes of direction).

**Figure 5.**
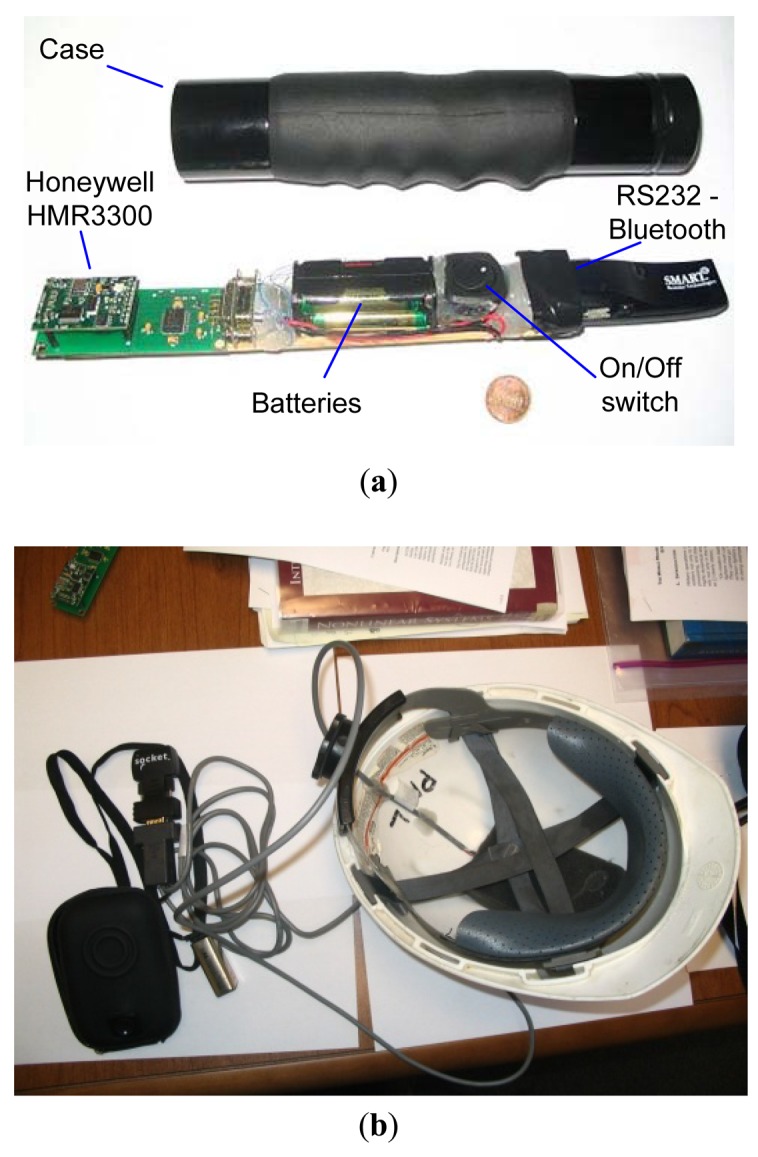
Prototypes built to conduct preliminary experimentation on the pointing and marking system (**a**) Handheld pointer; (**b**) Electronic compass in line with the helmet orientation (forehead) and wired (RS232) to rs232-bluetooth converter powered with a 12 V battery.

One of the key components that makes the above proposed system possible is the inclusion of electronic compass technology that allows to both provide high level accuracy of 0.5° with a resolution 0.1°, (Figure 4), and to generate precise readings with a low variance between 0.0025 and 0.01 degrees (Figure 5), which is the key element to control pointing drift and keep it stationary/predictable. Furthermore, electronic compass technology is able to maintain reliable compass readings even in the presence of transient magnetic fields.

Several pointing prototypes have been implemented and tested successfully (Figure 5). Advances in the precision and accuracy of location and orientation, obtained through technologies from companies like Honeywell and Raytheon, and the advent of more precise outdoor location systems allow us to expect favorable acceptance of systems like the one proposed in this document. Especially, considering that the precision and accuracy required by the FR are relative to human-perception. We envision that issues regarding the drift in pointing can be addressed in a relatively flawless manner by providing the FR with continuous near-real-time awareness (visual or audio-based feedback) of the objects being pointed to.

One of the most important aspects to evaluate from the system is pointing error including the pointing errors between satellite-borne and free-space optical communication systems. This error depends on the error the underlying technological components add to it; the GPS and the compass errors. Compass errors in particular can be of a challenge as environmental effects such as proximity to a strong magnetic field, aircraft engines, and higher concentrations of iron in the soil. These magnetic variations magnetic variation can mean a difference of up to 4 or 5 degrees in some places and in a chaotic environment can lead to a failed rescue mission. GPS errors on the other hand are accurate to just 1 percent of a bit time. This is approximately 10 billionths of a second (10 nanoseconds). Given that the GPS microwave signals travel at the speed of light, this equates to an error of about 3 meters. And can therefore be fatal to the rescue mission. Since standard GPS cannot determine position to greater than 3-metre accuracy more sophisticated GPS receivers are used by the military. Other errors arise because of atmospheric disturbances that distort the signals before they even reach a receiver. Reflections from buildings and other large, solid objects can lead to GPS accuracy problems too. There may also be problems with the time-keeping accuracy and the data onboard a particular satellite. These accuracy problems are circumvented by GPS receivers which endeavor to lock on to more than three satellites to get consistent data [11].

**Figure 6.**
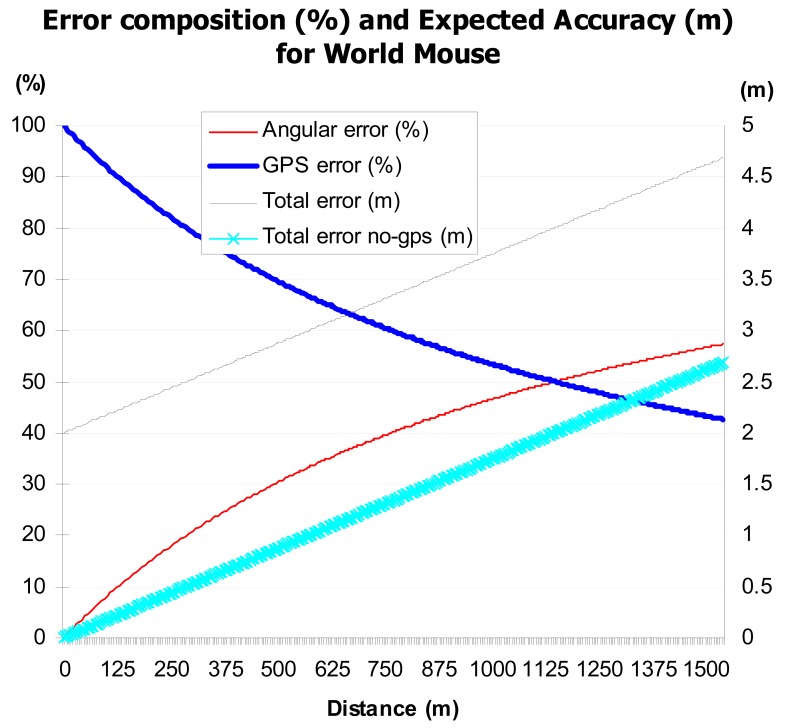
Error composition and impact. Percentage errors mapped on primary Y-axis, and total errors mapped on secondary Y-axis.

As shown in Figure 6, as the distance from the pointer to the target increases the error is increasingly affected by the angular error, *i.e.*, the compass error. On the other hand, as the distance decreases, the error is predominantly of GPS nature.

Some key results obtained for pointing were: (a) the system allows FRs incorporate stable/predictable drift in a natural manner; and (b) verification/validation of electronic compass's resiliency to transient magnetic interference, so that selecting objects in the immediate environment exhibited reliability ≥98%.

The remaining components of the system prototype were:

- Dell Axim PDA (WiFi + Bluetooth),
- Windows Mobile 5.0,
- C# Programming language,
- Generic GPS flash card,
- Microsoft Speech Server,
- In-house Google maps-based 3D model,
- Pointer-based vertical and horizontal gestures, and
- In-house developed 3D collision detection engine utilizing a traditional binary search tree technique for ray-target collision detection.

**Figure 7.**
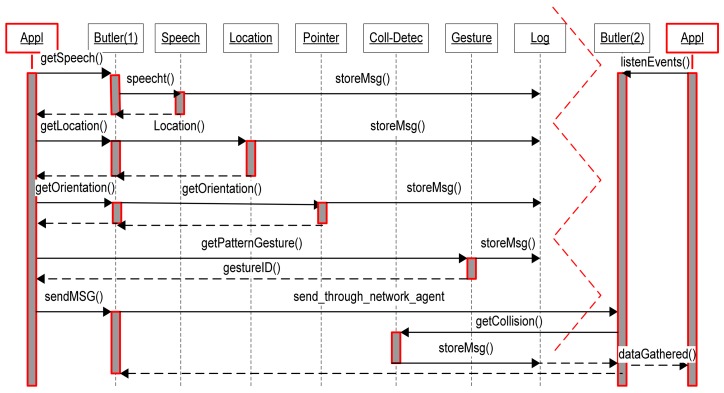
UML diagram of a query of a communication via the environment system.

All the components of the system were assembled with the agent-based architecture illustrated in Figure 7. The basic idea is that for every component there is a software agent which gathers data from the corresponding component by means of a pre-defined logical of physical gate. For example, the pointer or the GPS unit are configured to output their data through a serial port, then, the corresponding software agents will catch or fetch, depending on the communication protocol, the data sent by the sensor. A typical process for the system would be as follows. When an utterance is detected by the listener (speech recognition input service available to the application), it is sent to the *speech recognition service*/*agent*. The *speech recognition service* will respond indicating the status of the recognition task. At time intervals of length T seconds (parameter to be determined after experimentation), the *butler* (an agent which coordinates and controls the activities of the others agents in a node/device) collects the sequence of readings from the electronic compass gathered by the *orientation service*. Then it sends this information (treated as a set of vectors) to the *gesture pattern recognition service* to find matches with gestures defined by the user. After these processes are performed, the *application* (or the butler) requests the *location service* to inform the FR's location. When the location data are available, the *butler* and/or the *application* will be able to locate where in the 3D model the origin and direction of a ray is coming from. Lastly, all of the transactions will be registered in the *log service*.

The system prototype described in the previous paragraphs was tested by users from the Defense Advanced Research Projects Agency (DARPA). Test-users pointed to large objects from the physical surroundings and retrieved pictures of those objects (buildings) on the screen of a handheld device. This activity required minimal training and preparation; between 5 and 10 minutes were required by two DARPA officers who had no previous knowledge about the prototype to learn and operate it successfully, which demonstrate the natural and intuitive characteristics of the system. DARPA users reported their experience in a descriptive and qualitative manner which was instrumental in the next generation of the ULTRA-VIS program.

The system prototype was further tested by a group of 10 people from Applied Research Associates, Inc. in Champaign, IL. The testing group included Division and Sector Managers, engineers and administrative staff to guarantee variety in the testing effort. The testing was framed on qualitative methodology with emphasis on user perception and satisfaction in pointing and marking tasks. The users were presented with the survey presented in Table 1.

The qualitative testing threw some important results. While 80% of the people answered option (d) to question 1 while 20% selected (c). Regarding pointing and marking, addressed in question 2; 90% answered option (c) and 10% option (a). All answers for question 3 were (a). Finally, 100% of the surveyed people selected answer (a) for question 4, stating the system as easy to use.

## 4. Discussion

The technology presented in this article enables FRs to reference objects and peers in the physical world via 3D Terrain Models and other existing technologies. The strength of this system is certainly on the natural and intuitive interface that enhances the communication through the environment. The motivation to further develop this technology is that there is no technology adequate for the vector (user, task, and context) existing on inhospitable and highly dynamic disaster relief environments: users simply cannot waste time using GIS-based applications if we can provide direct capability to communicate via the environment.

A team of FRs using these technologies will be able to interact with the directly perceived environment, rather than more traditional heads down digital representations of target objects. The proposed approach is radically different from current GIS-based applications, which use artificial space (e.g., map) to mediate interaction within a group of field FRs. The performance of a FR however is expected to significantly improve due to suppression of the real-world-to-model mapping layers. Suppression of the mapping layers will (a) allow for less context switching (*i.e.*, from real world to its representation and *vice-versa*) and consequently lead to a lower error rate in referencing and land marking so the FR team can act faster to successfully complete their coordination tasks. This observation stems from studies using automated emergency response systems with other either manual or hybrid systems [12]; and (b) allow faster access to critical information such as buildings under an immediate threat; and (c) allow faster command and control decision given near-real-time pointing information send back from FRs in the field.

Results presented in this article are encouraging to continue and develop further research on a system to enable communication through the environment. COTS technology advances very fast, providing new and better alternatives for most of the components of the integrated system. Even the system has not been proven and tested in real scenarios, we will improve, advance, and test this prototype system in the near future with the support and collaboration of the Illinois Fire Service Institute, located in Urbana, IL, to test the interest and approval in pursuing an evaluation process for this approach in various training scenarios. Further work aims to explore real interest from FRs community in a system like the one presented in this article.

The research presented in this paper is part of a framework proposed by the authors for FRs to cooperatively improve situational awareness between them. For example, FRs would be able to detect a peers' location while interacting by voice with those peers. This approach is completely opposite to predominant Augmented Reality and GIS-based research, which focuses on graphics generated by the computer. In other words, it is an augmented reality approach where user interaction is mainly based on hand and arms gestures and voice recognition. The overall performance is expected to significantly improve due to fewer location errors, better overall focus, and reduced communication time/effort between FRs if compared to GIS-based navigation systems. Direct referencing as proposed in our study has therefore the potential to be more effective than symbolically referencing physical objects. The symbolic layer currently utilized in GIS systems adds an artificial symbolic layer to the interaction between the user and his/her immediate environment, which slows down overall performance. Removing all the activities that are involved in this layer should improve the referencing process (e.g., the coordination of location and movements among the members of an emergency response team, where every second or error counts). For FRs operating in inhospitable, chaotic, and highly dynamic environments, we argue both that no symbolic layer is required for referencing-based activities, and that existing and new technology breakthroughs allow us to design such system interfaces.

## 5. Related Work

The system presented in this article is designed to provide new capabilities for FRs. The technology incorporates concepts and principles from virtual reality [13–15] and augmented reality (AR). However, most related research in AR focus on commercial or everyday life in non-hostile and non-chaotic environments [16–18]. The characteristics that differentiate this research from AR related research is its aim to function with an analog rather than a digital interface. For the FR and the technology to be able to focus on analog gestures are unique in cognitive terms and help improve to deal with the demands of situational awareness in a chaotic environment.

During the last few years we have witnessed the attention that both the scientific and the technological communities have put on the situational awareness domain. For example the System for Wearable Audio Navigation (SWAN) from the Sonification Laboratory at Georgia Institute of Technology providing situational awareness while requiring minimal visual attention from the user [19–21]. SWAN is a technology with emphasis on helping people with vision disabilities [22]. The framework to enable communication through the environment proposed in this article will benefit from capabilities like SWAN and visual-based collaboration [23], which improve situational awareness for the busy field first responder. Conversely, research such as Emergency Automated Response System [EARS] that has been done by some researchers may further enhance the necessity for better combination of technology and the human senses. Relying on technology alone does not sufficiently advance the success of rescue missions but rather a solid platform between the human and modern technology will be required in the future [24–28].

## 6. Conclusions

The system proposed in this article requires the integration on several technologies. This article demonstrates that COTS technology can support all the functional components required to achieve the proposed system. There are several technological choices to implement the augmented reality-based annotation and retrieval mechanisms described here. For example, while the more suitable option to augment vision capability might be the use of see-through head ups displays, current camera-enabled cell phones and tablets, with 3-axis accelerometers and compass, can still provide basic functionality.

Pointing drift is not a relevant problem for the emergency response application given the cognitive skills we as human beings embrace. For systems where a human being is not part of the pointing drift correction mechanism, such drift becomes a serious deterrent for the success of the system applicability.

This work aims to enable communication through the environment for mobile users that usually have incomplete information and have limited processing capability while attending very demanding, and sometimes unpredictable tasks. In this environment, we argue a natural and intuitive interface involving basic and simple cognitive processing capabilities is more suitable for FRs. This interface is predominantly based on analog communication; pictorial/iconic information instead of text, gestures, and tactile media. Performance is expected to significantly improve due to less error, focus, and time invested than in navigation through a GIS-based application, therefore the referencing is not symbolic but directed to the physical object.

The encouraging results obtained demonstrate this applied research has the potential to enable communication through the environment for FRs operating in highly dynamical and inhospitable disaster relief environments.

## Figures and Tables

**Table 1.** Prototype qualitative evaluation.

**Question**	**Possible answers**
1–How would you describe the prototype regarding how difficult is it to be learned to used	(a) Difficult (b) Requires effort (c) Not a big deal (d) Easy to learn
2–How would you say the system performs in pointing and marking the surrounding buildings?	(a) Bad (b) Regular (c) Good (d) Excellent
3–Is the system's response time adequate?	(a) Yes (b) No
4–How would you describe the system's usability	(a) Easy to use (b) Regular (c) Some difficulty (d) Difficult
